# Membrane staining and phospholipid tracking in Pseudomonas aeruginosa PAO1 using the phosphatidylcholine mimic propargyl-choline

**DOI:** 10.1099/acmi.0.000690.v3

**Published:** 2024-11-28

**Authors:** Chris L.B. Graham, Jack Bryant, David I. Roper, Manuel Banzhaf

**Affiliations:** 1 School of Life Sciences, University of Warwick, Coventry, UK; 2 School of Biosciences, University of Birmingham, Birmingham, UK; 3 School of Life Sciences, University of Nottingham, Nottingham, UK; 4 Newcastle University Biosciences Institute, Newcastle University, Newcastle, UK

**Keywords:** Cy3, Dye, lipid tracking, membrane label, propargyl-choline, *Pseudomonas aeruginosa*, phosphatidylcholine

## Abstract

The use of membrane-specific dyes for *in vivo* fluorescent microscopy is commonplace. However, most of these reagents are non-specific and cannot track specific lipid species movement, instead often acting as non-covalent lipid-associated probes or requiring the uptake of whole lipids and acyl tails into the membrane. This issue has been solved in eukaryotic cell biology by the use of click-chemistry-liable phospholipid headgroup pulse labels. Here, we describe a method for *in vivo* phospholipid labelling by fluorescent imaging in *Pseudomonas aeruginosa* using a phosphatidylcholine mimic, ‘propargyl-choline’ (PCho). This click-chemistry-liable headgroup mimic is visible by microscopy and allows the covalent labelling of lipids. Fluorescence of the cell membranes, visible in heterogeneous patches, is dependent on PCho concentration and is localized in the membrane fraction of cells, demonstrating that it is suitable for membrane labelling and cell imaging.

**Impact Statement**

*Pseudomonas aeruginosa* is an opportunistic pathogen. Pathogenicity and antibiotic resistance of the organism can be partly attributed to the presence of phosphatidylcholine (PC) lipids and more broadly the cell envelope. In 2019, more than 82 000 people died due to *P. aeruginosa* strains with resistance to one or more antibiotic treatments [25]. To enable better study and understanding of *Pseudomonas* sp. lipids, we describe an *in vivo* method to label *P. aeruginosa* PC lipids and describe their subsequent visualization by click-labelling and fluorescent microscopy. The phospholipid headgroup mimic propargyl-choline (PCho), substituting for a PC headgroup, has previously been used in mammals as a click-able microscopy label for use in membrane stability assays in engineered bacteria. Here, we show its use in ‘WT’ bacterial cells, as a method to visualize the movements and localization of membranes, similar to FM4-64 and applicable in situations where the tracking of a specifically labelled membrane lipid is useful. The ability to image a lipid mimic such as PCho in a model species such as *P. aeruginosa* using PCho in bacteria could also, as in eukaryotes, provide insight into lipid-related organizations, growth and replication stages of bacteria in general not yet touched on.

## Data Summary

The microscope images, code and GIFs of the lipid movement can all be found on the associated Figshare article available at https://doi.org/10.6084/m9.figshare.27292464.v1.

Data S1 – GIF of Cy3-propargyl lipid movement multiple cells.

Data S2 – GIF of Cy3-propargyl lipid movement individual cell (tracked).

Fig. S1, available in the online version of this article – Concentrations of propargyl-choline and effect on image quality and survival.

The authors confirm all supporting data, code and protocols have been provided within the article or through supplementary data files.

## Introduction

The insertion and maintenance of lipids in the inner membrane and inner leaflet of the outer membrane of Gram-negative bacteria are not yet fully understood. Whilst labelling of cells with lipid probes has revealed lipid raft localization, cardiolipin localization [1,3] and changes in phospholipid abundance over time, few methods can track covalently modified lipid movement. Fluorescence-labelling techniques for microscopy typically monitor lipid movement and localization using probes able to detect specific lipid headgroups [2] or by labelling the lipid tail [4]. The phosphatidylcholine (PC) mimic, propargyl-choline (PCho), has recently been used to label phospholipids in *Escherichia coli* cells, which were modified to include PC metabolism through addition of phosphatidylcholine synthase (pcs), a condensation enzyme which can produce phosphatidylcholine using free choline and CDP-acylglycerol. PCho-labelled cells were suggested to have PCho in the inner and outer membranes; however, PC biosynthesis is not native to *E. coli*, and the study did not focus on the labelling and imaging processes [5]. Here, we chose to study the distribution of the PC mimic in WT *Pseudomonas aeruginosa PAO1*, as this organism natively has PC in the cell envelope.

*P. aeruginosa* is an opportunistic pathogen, with pathogenicity and antibiotic resistance associated with its cell envelope structure [5,6]. PC has been shown to be required for efficient infection, acting as an inflammation facilitator and precursor to lung damage [7]. Similar pathogens also scavenge PC from the host [8], which may play a role in growth, and PC has been shown to be required for cytotoxin production in related *Pseudomonas* sp. [9]. Therefore, labelling technologies that enable the analysis of exchanges and dynamics of lipids, especially PC, would be a valuable tool.

Mimics for PC have been developed for mammalian studies. In the mammalian system, PC metabolism can be directly supplemented with soluble choline mimics, which do not affect other aspects of core cell metabolism [10]. PCho has yet to be used in WT Gram-negative bacteria for PC pulse-chase labelling. However, the capacity to label, image and track phospholipids in Gram-negative bacteria would enable this process of lipid insertion and dynamics to be studied simultaneously with peptidoglycan biogenesis through the simultaneous use of fluorescent d-amino acid mimics to address questions of how these processes are coordinated in the cell [11]. This is possible by taking advantage of the pcs pathway in *P. aeruginosa* [5,7], to allow for the uptake of non-native PCho as an alternative to the PCho donated to cytidine diphosphate diacylglycerol by pcs to form PC.

Therefore, in this study, we establish the use of PCho [10] to determine the localization of the phospholipid PC in *P. aeruginosa* after chemical crosslinking to an azide group. We found PCho to be of similar technical use to existing membrane labels such as FM464, however with the potential for tracking this specific lipid species distribution and behaviour rather than displaying an affinity for detecting or binding to lipids in general [12]. Using PCho, a soluble headgroup rather than lipid tail addition, also removes the need for membrane perturbation when using full phospholipid addition [13] or the use of mutant cells with alternative enzyme pathways to WT [5].

## Methods

### Imaging of fluorescence in *P. aeruginosa*

Strains were streaked from glycerol stocks onto LB agar plates and incubated at 37 °C overnight. One PA colony was grown overnight at 37 °C, at 180 r.p.m. in 2 ml LB. The following morning, a 1/10 dilution of the samples was then grown at 37 °C, 180 r.p.m. until the samples had all reached an OD_600_ of 0.3. One per cent of agarose in PBS was heated in a microwave until piping hot. Microscope slides were topped with a solution of 1% agarose in PBS, which was flattened with a coverslip and left to cool. The coverslip was removed, and 10 µl of the sample was added to the slide; the coverslip was placed on top to spread the sample across the slide. Samples were then analysed using confocal microscopy specifically to identify the fluorescence, with corresponding filters dependent on the expected fluorescence. Cy3-PCho fluorescence was detected using a TXR filter set on a Leica DMi8 confocal microscope. The resulting images were taken in clusters of 15 across all the samples at random to reduce bias and allow for quantitative cell measurements.

### Analysis of fluorescence localization

Images were imported into Leica Image File (LIF)s or Tagged Image File (TIF) libraries, which were then analysed by MicrobeJ software to determine cell contouring and maxima points. The points of increased fluorescence to the background were then tracked across the cell and mapped per individual point across thousands of cells, dependent on cell length. The points were then mapped for each strain. Automatic cellular counting and size determination by MicrobeJ [14] and BactMAP [15] were allowed for quantitative analysis. Fluorescent points were tracked using custom tolerance and intensity filters maintained throughout the study. Scripts and conditions for image analysis are attached in the Supplementary material file.

### PCho click-labelling

**Fig. 1.**
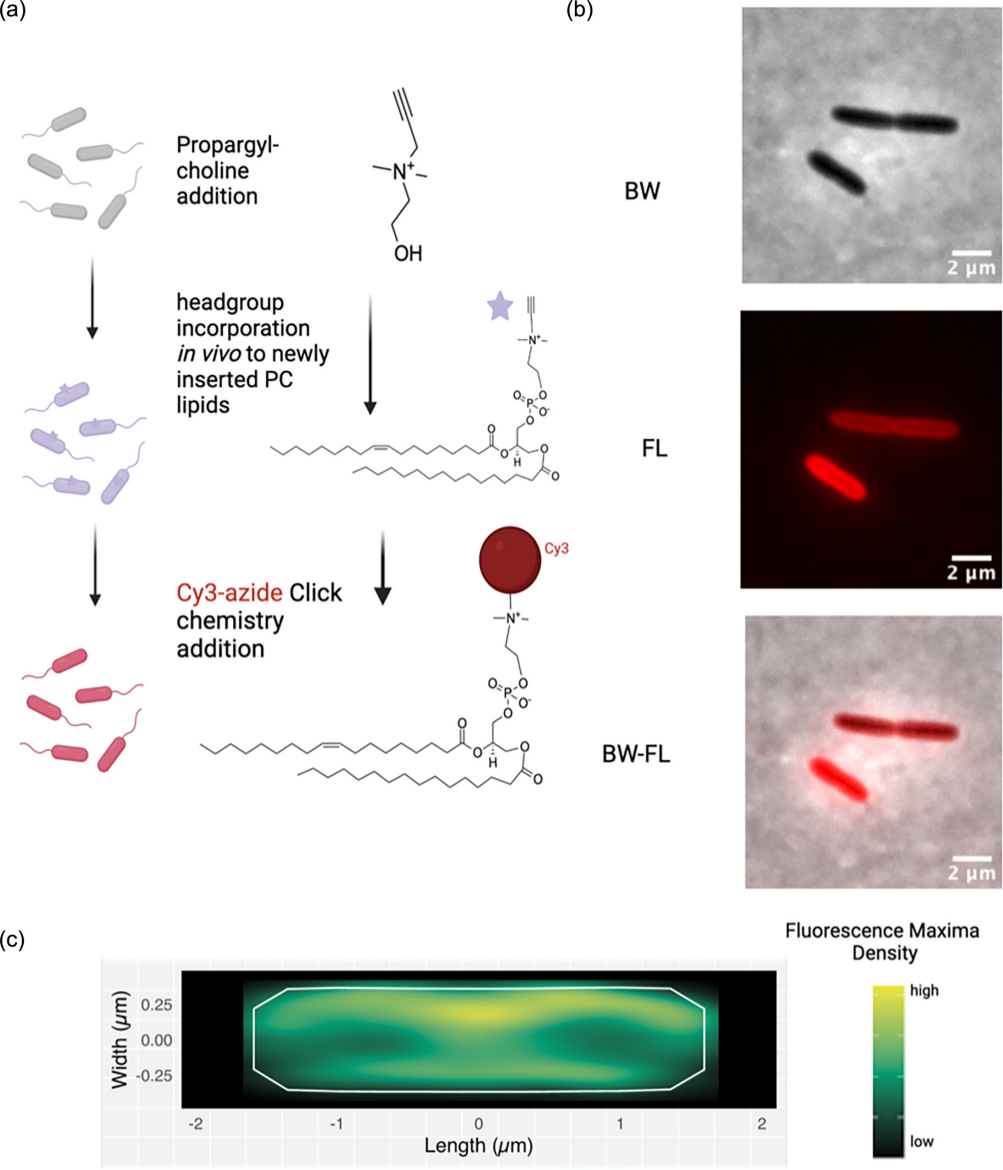
PC lipid insertion can be visualized by PCho. (**a)** PCho mimics PC and can allow for the PC precursor cytidine diphosphate diacylglycerol to be attached to PCho through the pcs pathway, and the propargyl group can be labelled by click chemistry. (**b)** Membrane localization of PCho-cy3 fluorescence (100 µM). (**c)** Average localization of cell fluorescence maxima 1119 cells, 0.1 OD exponential phase by BACTmap [15].

**Fig. 2.**
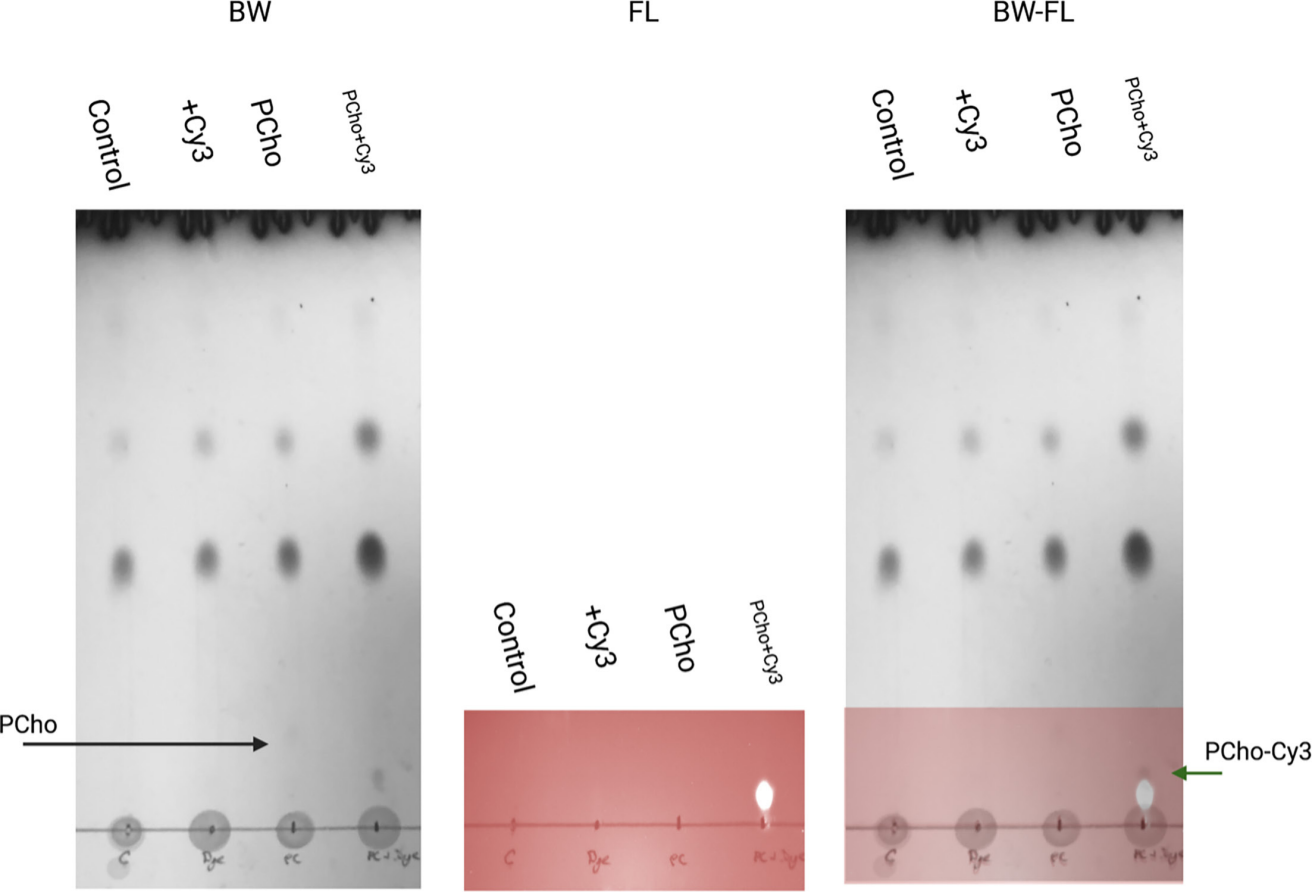
Insertion of PCho and Cy3 in *P. aeruginosa* membranes. TLC plate of *P. aeruginosa PAO1* lipid fractions. All cells were subject to conditions used for click chemistry described in Methods. Control – WT *P. aeruginosa* lipid extract, +PCho 100 µM PCho addition, +Cy3 with Cy3 addition, PCho and Cy3 and both PCho and Cy3 added. BW – visible light photo of stained TLC plate. FL – Cy3 fluorescence indicating Cy3 lipid attachment to lipid fractions. Black arrow – propargyl-choline-labelled lipid peak. Green arrow – Cy3-propargyl-choline-lipid peak.

*P. aeruginosa* PAO1cells were grown to 0.1 OD and then incubated with alternative concentrations of PCho (dissolved in DMSO) (1–2800 μM) for 5 min. These cells were then concentrated by centrifugation for 10 min at 5000 ***g*** and (Figure 1/3/4) fixed by 4% paraformaldehyde PBS for 30 min; however, this is not necessary for labelling (Figure 2/5). Cells were washed by centrifugation at 5000 ***g*** by pelleting and resuspended in 100 mM Tris-HCL, pH 8.8, 1 mM CuS04 50 µl and ascorbic acid 0.1 M. The cells were reacted with 100 µM Cy3-azide, for a click-chemistry reaction, then after 30 min at room temperature washed with TBS 1% Tween solution by centrifugation four times to remove the fluorescent azide remaining before imaging.

### TLC and lipid extraction

Lipid extraction used the Folch method [16] of lipid extraction, with 1 : 2 : 1 chloroform:methanol:water at 55 °C for 30 min, and vortexing, followed by extraction of the chloroform lipid phase after centrifugation. The TLC was conducted on 60A Sepharose 254 nm TLC plates, using 65 : 25 : 10 chloroform, ethanol and acetic acid. The TLC plate fluorescence was recorded using Cy3 fluorimetric TXR filters on a 5× Zeiss Axio zoom microscope and posed adjacently.

### Tracking lipid foci

Trackmatev6.02, implemented through a FIJI package, was used to identify foci and track their movement over time [17]. Cells observed in octanol 1 mM were compared to H_2_0 and imaged at 100 ms intervals using TXR filters. Trackmate parameters: Cell threshold 60,000, foci diameter 0.1 µm, linking distance 0.3 µm, gap closing 0.3 µm and Simple LAP tracking, LoG detector and subpixel localization=True.

## Results

### The PC headgroup mimic PCho localizes to *Pseudomonas* membranes

To determine the efficacy of PCho as a membrane-localizing PC mimic, the compound was incubated with *P. aeruginosa PAO1* cells during exponential growth for 5 min. This growth period equates to ∼0.25 of the generation time in these growth conditions [18]. Cells were then fixed and labelled with a fluorescent Cy3-azide, as established in mammalian cells [10]. The cells were also labelled for teichoic acid as previously described [19] (Fig. 1a). Cells incubated with PCho that underwent click-labelling demonstrated fluorescence at the membrane after click-labelling for 5 min. This indicates that the lipid head group mimics are localized to the membrane specifically (Fig. 1b).

We then optimized PCho staining across a variety of concentrations (Fig. S1), with 100 µM being the optimum concentration for membrane visualization. However, averaging of fluorescence distribution using BACTmap software [15] (Fig. 1c) revealed no specific increase in fluorescence at any region except for the membrane and membranous cell division site. Titration of PCho revealed no fluorescence above the background in the absence of PCho and visible fluorescence in cells from 1 µm to 100 µM PCho (Fig. S1b).

The optimum insertion time was 5 min at 100 µM PCho, which was sufficient to visualize membranes after washing. However, lower concentrations were also sufficient for visualization (Fig. S1b). To determine whether PCho, and its storage buffer DMSO had a detrimental effect on growth, we measured the growth cycles of cells with higher PCho concentrations (Fig. S1b). Only concentrations above 150 µm PCho, corresponding to 2.5% DMSO, affected the growth rate. This effect was marginal and insignificant during the growth phase. This indicated that at the PCho concentrations used for microscopy (1 µm–100 µM) the growth defect was insignificant.

We then confirmed the incorporation of PCho into the membrane through the pcs reaction as a PC facsimile by TLC of the lipid fraction prepared from whole *P. aeruginosa PAO1* cells after click-chemistry treatment. The fluorescent headgroup of PCho-cy3 was only incorporated in cells that had both been labelled with PCho and Cy3 after click-chemistry labelling. This indicates that PCho was incorporated into the lipid fraction of cells to facilitate lipid labelling (Fig. 2). Phosphomolybdic acid staining for lipid species revealed an additional spot by TLC for cells without Cy3 addition. This could potentially represent an unlabelled PCho PC lipid mimics that adopted PCho into lipids by the pcs pathway but were not labelled by Cy3 (black line).

**Fig. 3.**
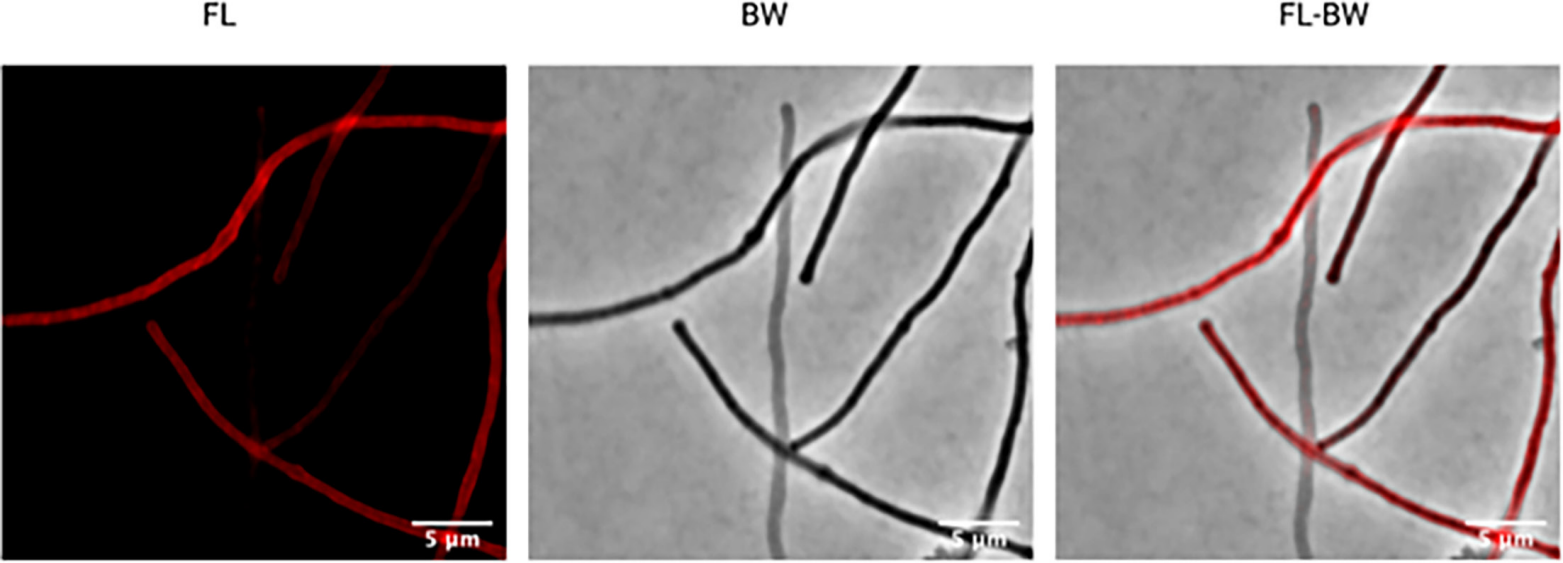
Aztreonam elongated cells show localized fluorescent incorporation. Representative figure of banding in elongated cells. BW – confocal, FL – fluorescent wavelength, BW-FL – merged confocal fluorescence images. All images were brightness and contrast adjusted manually for the highest-level detail observable in fluorescence channels. 5 min incubation at 100 µM PCho.

To show the use of PCho labelling in antibiotically challenged cells and investigate the uniformity of fluorescence of the cy3-PCho lipids, we labelled elongated cells where differences could be more easily shown due to potential lipid concentration differences. *P. aeruginosa* cell elongation was achieved by exposure to the *β*-lactam antibiotic aztreonam, which is highly selective for inhibition of cell division-associated PBP3 (FtsI) [20]. We hypothesized that if new PC is incorporated into the membrane at distinct sites, then this could be visible in elongated cells. We did not see any quantifiable difference or reasoning for the differences observed but found that labelled cells do have regions of visibly higher intensity fluorescence and lower intensity fluorescence (Fig. 3). If this could be quantified or shown in other scenarios, this may lend PCho click-chemistry labelling extra utility.

### PCho-Cy3 fluorescence has a similar localization pattern to FM-464X

**Fig. 4.**
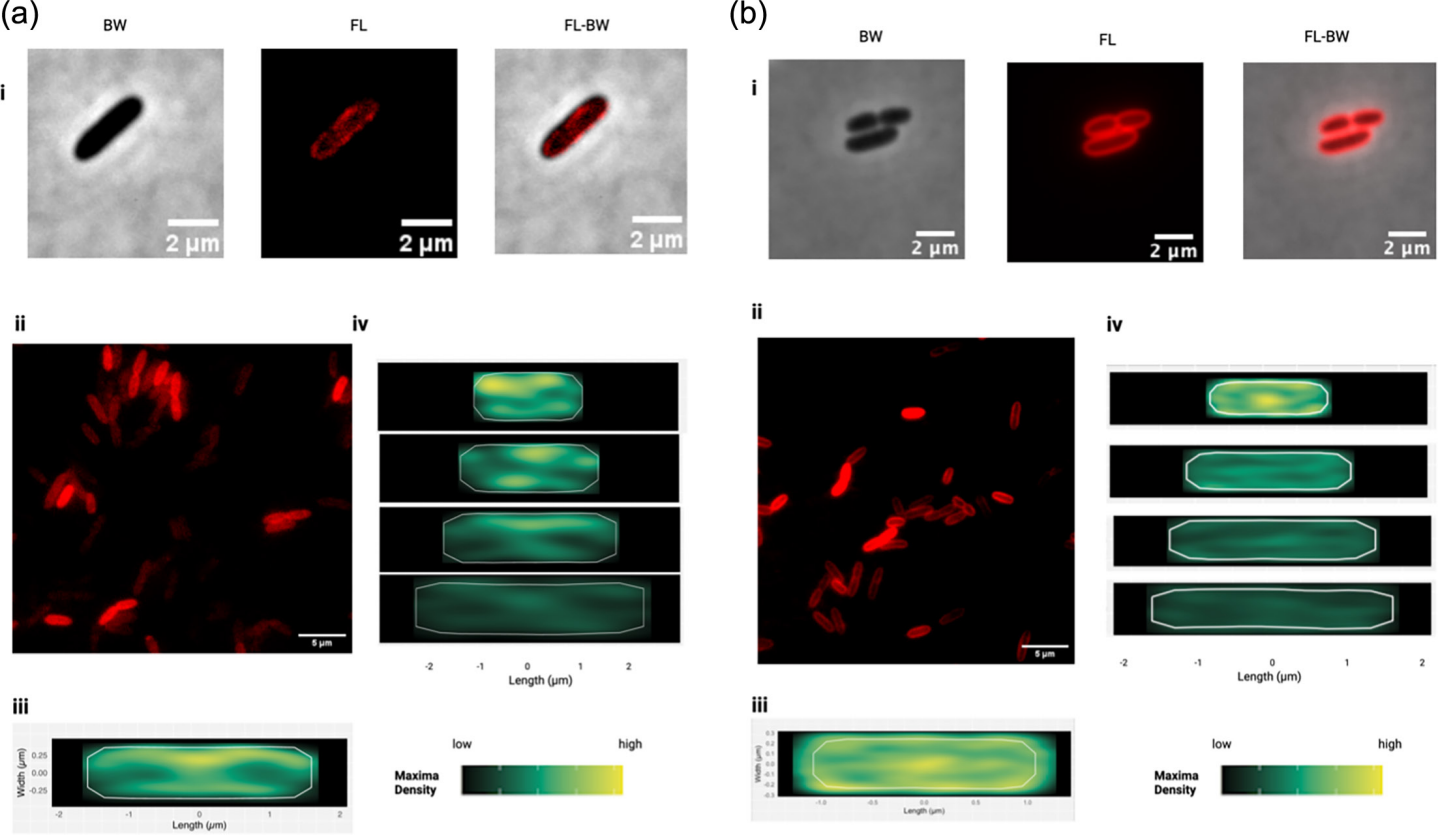
Membrane localization and preference of PCho-labelled cells. (**a)** Localization of Cy3 fluorescence in PCho time-pulsed cells after 5-min incubation with 100 µM PCho on individual cell i, groups of cells ii, and analysed as a population of 1119 cells across length classes iii/iv by BACTmap. (**b)** FM4-64X localization on individual cells i, groups of cells ii, and analysed as a population of 224 cells across length classes by BACTmap iii/iv.

To evaluate Cy3-mediated fluorescence of labelled PCho as a general membrane stain, we compared it with a widely used membrane localization method, the membrane dye FM-464X, a fixable alternative to FM4-64 [21] (Fig. 4). We found that the FM-464X membrane localization was a visually clearer membrane label than PCho (Fig. 4). However, fluorescence was heterogeneous on the cell level that were stained with either FM-464X or PCho. This suggests that differences in fluorescent intensity patterns between cells could be dependent on preparation/visual depth as indicated by maps of fluorescent maxima, which have a peak at the cell centre. However, heterogeneity in PCho-labelled cells also occurred at the membrane level. The heterogeneity of fluorescence within individual cells was suggestive of varying distribution of label incorporation, suggesting a real fluorescence heterogeneity among lipids.

### The PCho click-labelled lipids can track lipid movements

**Fig. 5.**
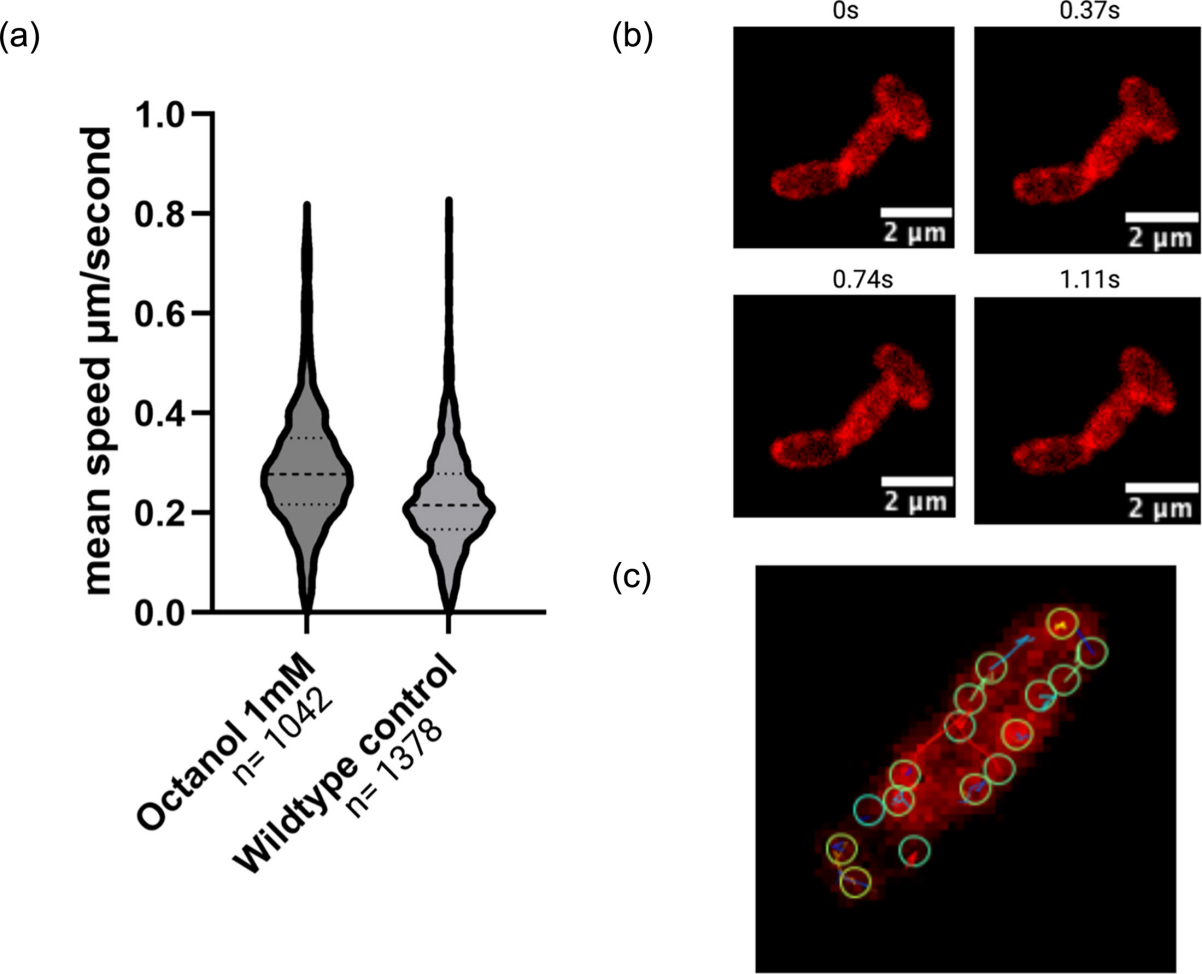
Use of PCho as a potential lipid domain visualization tool. (**a)** Trackmate [17] mean track speed µm/s and fluorophore counts in a *P. aeruginosa* PAO1 PCho and Cy3-labelled cells. T-test difference in population, *P*<0.0001. (**b)** 1.11 s timecourse of PCho-Cy3 fluorescence movement attached GIF in supplementary material showing fluorescence change over time in the periplasm. Red indicating fluorescence (Supplementary material). (**c)** 100 ms exposure Cy3-labelled PCho *P*. *aeruginosa* cell, Trackmate fluorescence domains capture and tracks shown.

Upon observing the heterogeneity of fluorescence, we hypothesized that the PCho labelling methodology could be used to track lipid domains in live cells. The Cy3 click-labelled PCho photobleached over time; however, the heterogeneous patterns of fluorescence localization changed over the course of milliseconds, independent of the bleaching effect. This suggested that the ~3% phospholipids that were potentially labelled could be changing location over the course of the imaging experiment. These groups of lipid particles could be tracked in ImageJ [22] using Trackmate [17] to give a population of potential lipid speeds over time. Lipid group track speeds revealed movement around a periplasmic track between 0.25 and 0.35 µm s^−1^, which is similar to the speed of these lipids in mammalian cells [23] (Fig. 5). Speed of group movement increased following exposure to 1 mM octanol, which is known to increase membrane fluidity [24] (Fig. 5a). This indicates that tracking the lipid particle groups is indicative of lipid movement within the membrane. These results suggest that regions of increased fluorescence labelling could indicate lipid microdomains and lipid movements. Therefore, this method could have potential uses in total internal reflection fluorescence (TIRF) microscopy as a means of studying lipid movement phenomena.

## Conclusion

In this work, we have adapted an existing technique for PC localization in eukaryotic membranes for use in WT bacterial cells. Pulse-labelling *P. aeruginosa* cells with PCho allowed for visualization of new PC mimic insertion in the cell membrane of living cells. (Fig. 1). We confirmed the insertion of the label using extracted lipids from *P. aeruginosa* PAO1, which revealed a fluorescently labelled group visualized by TLC, only when both Cy3-azide and PCho were present during labelling.

This visualization of a specific lipid directly through a soluble compound followed by click-labelling, as opposed to other lipid labels, which label lipids dependent on tail or have variant lipid affinities, allows for a specific and titratable bacterial membrane label with direct visualization of lipids, rather than affinity. Analogous mimic labels, which have a similar purpose for use in cell envelope studies, such as peptidoglycan labelling with fluorescent d-amino acid mimics, have in the past been used in fluorescence microscopy, allowing for significant advancements in the study of bacterial peptidoglycan [11], and so PCho labelling may have a similar function, especially in combination with other methods and azide label combinations. However, it should be noted that PCho incorporation has also been used for teichoic acid visualization, as choline is also used during teichoic acid synthesis [19]. Therefore, the choice of a species without teichoic acids, such as *P. aeruginosa*, is necessary to allow for a direct lipid visualization without potential crossover. Related species such as *Brucellus abortus* have been shown to use PC in their own membranes, after scavenging from the host eukaryotic cells [8]; therefore, a marker for PC where choline mimics are internalized and directly tracked in bacteria would be useful for understanding these processes and the nuances of pathogenicity more clearly.

In this study, having tested this in *P. aeruginosa* PAO1, we propose this PCho incorporation and visualization method as a phospholipid fluorescent labelling technique for compatible bacteria when other general labels are not suitable. We also propose that the covalent label may have further use due to its titratable nature, in single-molecule tracking techniques for the study of lipid dynamics in bacteria.

## Abbreviations

PC: phosphatidylcholine
PCho: propargyl-choline
pcs: phosphatidylcholine synthase
